# Prevalence, Disease Onset and Clinical Outcome in Arginase 1 Deficiency: Cross‐Border Surveillance in Germany, Austria, and Switzerland

**DOI:** 10.1002/jimd.70210

**Published:** 2026-06-07

**Authors:** Svenja Scharre, Annette L. Hess, Florian Gleich, Sven F. Garbade, Patrik Feyh, Daniela Karall, Anna Baghdasaryan, Martina Huemer, Julia B. Hennermann, Andreas Hahn, Martin Lindner, Gwendolyn Gramer, Thomas Luecke, Nils Kleinelanghorst, Johannes Haeberle, Amelie S. Lotz‐Havla, Georg F. Hoffmann, Juergen G. Okun, Ulrike Muetze, Stefan Kölker

**Affiliations:** ^1^ Department I, Division of Pediatric Neurology and Metabolic Medicine Heidelberg University, Medical Faculty Heidelberg, Center for Pediatric and Adolescent Medicine Heidelberg Germany; ^2^ Division of Metabolic Diagnostics and Newborn Screening Heidelberg University, Medical Faculty Heidelberg Heidelberg Germany; ^3^ Department for Pediatric and Adolescent Medicine, Clinic for Pediatrics I Medical University of Innsbruck Innsbruck Austria; ^4^ Division of General Pediatrics, Department of Pediatrics and Adolescent Medicine Medical University of Graz Graz Austria; ^5^ Division of Metabolism and Children's Research Centre University Children's Hospital Zurich, University of Zurich Zurich Switzerland; ^6^ Department of Paediatrics LKH Bregenz Bregenz Austria; ^7^ Competence Area Healthcare and Nursing Vorarlberg University of Applied Sciences Dornbirn Austria; ^8^ Villa Metabolica, Center for Pediatric and Adolescent Medicine, University Medical Center Mainz Mainz Germany; ^9^ Department of Pediatric Neurology and Center for Rare Diseases University Hospital Giessen Giessen Germany; ^10^ University Hospital Frankfurt, Center for Pediatric and Adolescent Medicine Department of Paediatric Neurology Frankfurt Germany; ^11^ Department for Inborn Metabolic Diseases University Children's Hospital, University Medical Center Hamburg‐Eppendorf Hamburg Germany; ^12^ Ruhr‐University Bochum, St. Josef‐Hospital, University Hospital of Pediatrics and Adolescent Medicine Department of Neuropediatrics and Social Pediatrics Bochum Germany; ^13^ Health Center for Children, Adolescents, and Young Adults, Medical Practice Doctores Dietz/Illerhaus, Focus Neuropediatrics Drensteinfurt Germany; ^14^ Dr. von Hauner Children's Hospital, LMU University Hospital, Ludwig Maximilian University Munich Munich Germany

**Keywords:** arginase 1 deficiency, Argininemia, epidemiology, phenotype, spastic paraplegia, urea cycle disorder

## Abstract

Arginase 1 deficiency (ARG1‐D) is an ultra‐rare urea cycle disorder characterized by progressive spastic paraplegia, developmental delay, epilepsy, and episodic hyperammonemia. Evidence on prevalence and clinical presentation is scarce. Therefore, epidemiology and the phenotypical spectrum were assessed in Germany, Austria, and Switzerland (DACH region). We conducted a questionnaire‐based, cross‐sectional study of confirmed ARG1‐D patients in the DACH region. Patients were stratified into early‐diagnosed (newborn screening [NBS] or high‐risk family screening [HR]) and diagnosed after symptom onset. We evaluated clinical, biochemical, and therapeutic characteristics of individuals with confirmed ARG1‐D. Epidemiological prevalence estimates were derived using national population data. A total of 20 patients were identified (Germany: 12, Austria: 7, Switzerland: 1). Eight were diagnosed early (NBS: 4, HR: 4) and 12 after symptom onset. Symptomatically diagnosed patients (median age 11 years) presented with a broad range of clinical manifestations, specifically progressive spastic paraplegia (67%), epilepsy (58%), dystonia (46%), developmental delay (58%), and hepatopathy (50%). Median age at diagnosis was 35 months in symptomatic patients versus 1 month in early‐diagnosed patients (*p* = 0.03). Estimated pediatric prevalence was 1:1042080 in the DACH region, with high regional differences. Hyperammonemia was reported in 72%. Enzyme therapy had been initiated in 21%; 2 patients underwent liver transplantation. ARG1‐D is a rare disease with a prevalence of approximately 1:1000000 individuals, and a complex and progressive clinical phenotype. Detection via NBS or HR allows early diagnosis and treatment initiation, potentially altering the clinical outcome.

**Abbreviations**
ALT: Alanine aminotransferase
ARG1(‐D): Arginase 1 (deficiency)
AST: Aspartate transaminase
CI: Confidence interval
CP: Cerebral palsy
DACH region: Germany (D), Austria (A), and Switzerland (CH)
HHH syndrome: Hyperornithinemia‐Hyperammonemia‐Homocitrullinuria syndrome
HR: High‐risk family screening
HSP: Hereditary spastic paraplegia
INR: International Normalized Ratio
NBS: Newborn screening

## 1 Introduction

Arginase 1 deficiency (ARG1‐D; OMIM #207800) is a rare autosomal recessive urea cycle disorder caused by biallelic pathogenic variants in *ARG1* leading to reduced arginase 1 activity and, finally, impaired ureagenesis [1]. Unlike individuals with severe disease variants of other urea cycle disorders, ARG1‐D does not commonly manifest with severe neonatal hyperammonemic decompensation. Characteristically, progressive spastic paraplegia, developmental delay, movement disorders, epilepsy, and episodic or chronic hyperammonemia emerge during infancy or childhood [2, 3]. The biochemical hallmark is persistent, often severe hyperargininemia. Recent studies have broadened the known phenotype, describing late‐onset or mild presentations initially classified as hereditary spastic paraplegia (HSP) or cerebral palsy [4, 5]. The global birth prevalence is estimated at 1.1–2.8 per million newborns [6, 7, 8], although ascertainment varies substantially. In Germany, Austria, and Switzerland, which form the so‐called DACH region, only the Austrian newborn screening (NBS) panel includes ARG1‐D as a target disease, while in Germany and Switzerland diagnosis of individuals with ARG1‐D is made after the manifestation of symptoms or in families with a previously identified index case.

Therapy consists of a protein‐restricted diet in combination with alternative pathway treatment with nitrogen scavengers, or liver transplantation in severe cases. Recently, pegzilarginase, the first enzyme therapy for ARG1‐D, has been approved by the European Medicines Agency as targeted therapy in affected individuals with ARG1‐D from 2 years of age [9, 10]. Early detection is crucial because neurodevelopmental injury in ARG1‐D may become irreversible once spasticity emerges and progresses [11, 12, 13].

This study provides the first cross‐national dataset of the DACH region aiming to estimate prevalence, disease onset, and clinical outcome, and to evaluate the impact of early diagnosis on outcome in individuals with ARG1‐D.

## 2 Methods

### 2.1 Study Design

In this cross‐sectional questionnaire‐based study across Germany, Austria, and Switzerland data on individuals with confirmed diagnosis of ARG1‐D was collected from February 2024 to December 2024. The study was approved by the local ethics committee of the main study centre Heidelberg, Germany (S‐675/2023, PI: S. Kölker). Study information was distributed by the “Surveillance Unit of Rare Neurological Disorders in Childhood” (*German*: Erhebung seltener neurologischer Erkrankungen im Kindesalter [ESNEK] [14]) to treating clinicians at metabolic and neuropediatric centres in the DACH region. Clinicians reported the number of patients with confirmed ARG1‐D (arm 1) and individuals with unexplained, progressive spastic syndrome (arm 2). They were asked to complete a pseudonymized, standardized case report form (arm 1 and 2; File S1), and send a dried blood spot card sample (arm 2). The study‐specific case report form contained information on medical history, clinical symptoms, diagnosis and treatment. For individuals in arm 2 dried blood spot testing for ARG1‐D (arginine concentration determined by tandem mass spectrometry) was offered.

### 2.2 Inclusion and Exclusion Criteria

Individuals with residency in Germany, Austria, or Switzerland were eligible for inclusion in arm 1 if ARG1‐D was confirmed biochemically, and enzymatically or genetically. Data collection in arm 1 was pseudonymized and therefore did not require written informed consent. Inclusion criteria for arm 2 comprised age ≤ 18 years, presence of etiologically unexplained progressive spastic paraplegia, and written informed consent from caregivers. Individuals were not eligible for arm 2 if genetic testing or amino acid quantification had already been performed.

### 2.3 Statistical Analysis

Patients were categorized as *early‐diagnosed*, if diagnostic mode was Newborn screening (NBS), or high‐risk family screening (HR); and as *symptomatically diagnosed* when diagnosed after onset of clinical manifestation by selective diagnostic work‐up. All statistical analyses were performed using “R”, a language for statistical computing and graphics (v4.3.1) [15]. Missing or implausible data were excluded case‐wise. Median and range described clinical data. For categorical variables, the absolute and relative frequencies were calculated. For continuous variables, the Wilcoxon rank sum test was used to compare medians between screened and symptomatically diagnosed individuals. Birth prevalence with 95% confidence intervals (CI) was calculated using the “epiR” package (v2.0.58) using national mid‐year population counts and birth rates [16, 17, 18]. Individuals above 18 years of age were not included in the prevalence calculation. A *p* value of 0.05 was considered statistically significant.

## 3 Results

### 3.1 Patient Cohort

**FIGURE 1:**
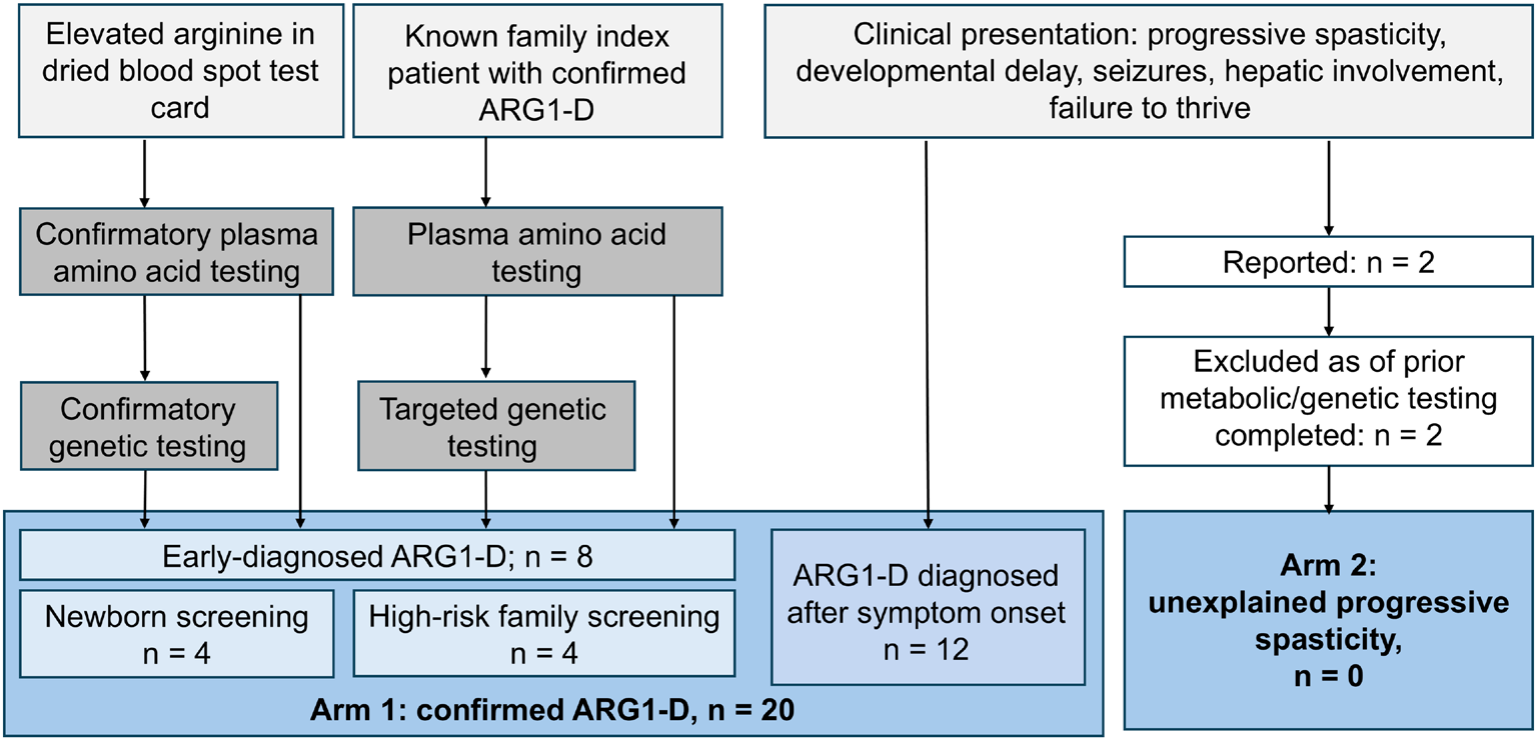
Flow of participants. In total, 20 patients with confirmed ARG1‐D were included in arm 1. Eight patients were identified early by NBS or high‐risk family screening. Twelve were diagnosed after onset of symptoms by selective diagnostic work‐up. No patient was eligible for inclusion in arm 2.

A total of 20 patients with confirmed ARG1‐D from 11 centers were included (arm 1; Germany: 12, Austria: 7, Switzerland: 1). The cohort consisted of 5 females and 15 males. Eight were identified early (40%) (NBS: 4, HR: 4), while 12 (60%) were diagnosed following the onset of symptoms (Figure 1). Two patients with unexplained progressive spastic paraplegia were reported for arm 2 but could not be included as genetic testing and amino acid analysis, respectively, had already been performed.

Median age [range] at data collection differed between early diagnosed (4 [< 1–21] years) and symptomatically diagnosed patients (11.5 [5–26] years; *p* = 0.03, Wilcoxon rank sum test). Known parental consanguinity was documented in 67% (8/12) of families with symptomatic diagnosed children and 43% (3/7) of early‐diagnosed families. Descriptive characteristics are displayed in Table 1.

**TABLE 1:** Descriptive characteristics of patients diagnosed after symptom onset versus early‐diagnosed patients with ARG1‐D.

	Patients diagnosed after symptom onset	Early‐diagnosed patients (NBS or high‐risk family screening)	Total
*N* (male/female)	12 (9/3)	8 (6/2)	20 (15/5)
Median age at last visit (range) [years]	11.5 (5; 26) Total *N* = 12	4 (< 1; 21) Total *N* = 8	10 (< 1; 26) Total *N* = 20
Consanguinity (*N*/Total *N*, %)	8/12, 67%	3/7, 43%	11/19, 58%
Diagnostic process			
Median age at diagnosis (range) [months]	35 (< 1; 144) Total *N* = 12	1 (0.1; 3) Total *N* = 8	2.75 (0.1; 144) Total *N* = 20
Biochemical confirmation (*N*/Total *N*, %)	10/12, 83%	7/8, 88%	17/20, 85%
Genetic confirmation (*N*/Total *N*, %)	12/12, 100%	7/8, 88%	19/20, 95%
Therapy			
Protein‐restricted diet (*N*/Total *N*, %)	11/12, 92%	8/8, 100%	19/20, 95%
Plus ammonia scavengers (*N*/Total *N*, %)	10/11, 91%	5/8, 63%	15/19, 79%
Plus Pegzilarginase (*N*/Total *N*, %)	4/11, 36%	0/8, 0%	4/19, 21%
Plus liver transplantation (*N*/Total *N*, %)	2/11, 18%	0/8, 0%	2/19, 11%
Neurological symptoms			
Spastic paraplegia (*N*/Total *N*, %)	8/12, 67%	0/8, 0%	8/20, 40%
Median age at start of paraplegia (range) [months]	30 (12; 192) Total *N* = 7	n. a.	30 (12; 192) Total *N* = 7
Need for use of orthopaedic devices (*N*/Total *N*, %)	8/10, 80%	0/7, 0%	8/17, 47%
Ataxia (*N*/Total *N*, %)	1/10, 10%	0/8, 0%	1/18, 6%
Tremor (*N*/Total *N*, %)	0/11, 0%	0/7, 0%	0/18, 0%
Muscular hypotonia (*N*/Total *N*, %)	3/12, 25%	0/7, 0%	3/19, 16%
Chorea (*N*/Total *N*, %)	0/10, 0%	0/8, 0%	0/18, 0%
Dystonia (*N*/Total *N*, %)	5/11, 46%	0/7, 0%	5/18, 28%
Reduced strength (*N*/Total *N*, %)	3/10, 30%	1/7, 14%	4/17, 24%
Epilepsy (N/Total N, %)	7/12, 58%	0/8, 0%	7/20, 35%
Median age at start of epilepsy (range) [months]	61 (7; 239) Total *N* = 6	n. a.	61 (7; 239) Total *N* = 6
Global developmental delay (*N*/Total *N*, %)	7/12, 58%	1/8, 13%	8/20, 40%
Developmental regression (*N*/Total *N*, %)	4/7, 57%	0/1, 0%	4/8, 50%
Failure to thrive (*N*/Total *N*, %)	3/12, 25%	0/8, 0%	3/20, 15%
Metabolic phenotype			
History of ≥ 1 hyperammonemic event (*N*/Total *N*, %)	9/11, 82%	4/7, 57%	13/18, 72%
Median max. NH_3_ (range) [μmol/L]	170 (90; 372) Total *N* = 9	123 (91; 321) Total *N* = 4	160 (90; 372) Total *N* = 13
Chronic hepatopathy (*N*/Total *N*, %)	6/12, 50%	2/8, 25%	8/20, 40%

*Note:* chronic hepatopathy is defined as hepatomegaly, elevated liver transaminases or elevated INR for longer than 3 months; hyperammonemic event is defined as ammonia level > 50 μmol/L.

### 3.2 Estimated Prevalence of ARG1‐D in Germany, Austria, and Switzerland

The estimated prevalence of ARG1‐D in childhood and adolescence was calculated based on the reported number of ARG1‐D patients under 18 years of age (16) and population data from Germany, Austria, and Switzerland. Assuming that all confirmed cases were reported, the prevalence of ARG1‐D in the DACH region accounted for 1:1042080 [95% CI 1:1041580–1:1042581] individuals with regional differences within the DACH region. The highest prevalence was calculated for Austria (1:315666 [1:315174–1:316159]), compared to Germany (1:1351172 [1:1350452–1:1351893]) and Switzerland (1:1583228 [1:1580763–1:1585696]).

### 3.3 Phenotypic Spectrum

Median age (range) at diagnosis was 35 (< 1–144) months in symptomatically diagnosed patients, and 1 (< 1–3) month in early‐diagnosed patients (*p* = 0.004, Wilcoxon rank sum test). Biochemical confirmation was obtained in 85% and genetic confirmation in 95% of all patients.

#### 3.3.1 Neurological Phenotype

At time of reporting, progressive spastic paraplegia, as the leading symptom of ARG1‐D, was present in eight patients (67%), all of them were diagnosed after onset of symptoms. Median age (range) at onset of spasticity was 30 (12–192) months. The use of orthopedic assistive devices was required in all of these cases. In addition to progressive spasticity, the clinical spectrum of symptomatically diagnosed individuals with ARG1‐D included global developmental delay in 58% (7/12), epilepsy in 58% (7/12), starting at the median (range) age of 61 (7–239) months, dystonia in 46% (5/11), and muscular hypotonia in 25% (3/12). Early‐diagnosed patients had minimal neurological symptoms (developmental delay in 1/8, and reduced muscle strength in 1/8).

#### 3.3.2 Metabolic and Hepatic Phenotype

A total of 13 patients (13/18; 72%) had already experienced at least one hyperammonemic event (9/11 symptomatically diagnosed, 4/7 early‐diagnosed patients), with a median (range) ammonia concentration of 170 (90–372) μmol/L (reference range < 50 μmol/L). The median maximum ammonia concentration did not differ between modes of diagnosis (symptomatically diagnosed: 170 [90–372] μmol/L, early‐diagnosed patients: 123 [91–321] μmol/L; *p* = 0.46, Wilcoxon rank sum test). Reported triggers for metabolic decompensation included infection such as gastroenteritis (2) and respiratory tract infection (2) or increased protein intake (1). Chronic hepatopathy (defined as hepatomegaly, elevated aspartate transaminase (AST) or alanine aminotransferase (ALT) or elevated International Normalized Ratio [INR] lasting longer than 3 months) was present in 50% (6/12) of symptomatic and 25% (2/8) of early‐diagnosed patients. 25% (3/12) of symptomatically diagnosed patients presented growth retardation or failure to thrive; a feature that was not reported in early‐diagnosed patients.

#### 3.3.3 Treatment Pattern

Almost all patients (19/20; 95%) followed a protein‐restricted diet, while one patient refused this diet. Nitrogen scavengers were used for pharmacotherapy in 79% of them (15/19), and enzyme therapy (pegzilarginase) in 21% (4/19). Pegzilarginase was used as monotherapy or in combination with dietary treatment or nitrogen scavengers. Two patients (10%) diagnosed after symptom onset underwent liver transplantation at 39 and 50 months, respectively (5 months after diagnosis in both patients). Both patients had spastic paraplegia and experienced at least one episode of hyperammonemia; one of the two patients presented with upper gastrointestinal bleeding and acute liver disease as the first symptoms at 16 months of age.

## 4 Discussion

This multinational cross‐sectional study provides the most comprehensive characterization to date of pediatric and adolescent ARG1‐D in the DACH region. We identified 20 confirmed cases, including 12 symptomatic and eight early‐diagnosed patients, revealing substantial differences in clinical presentation, neurological burden, and diagnostic timing.

### 4.1 Prevalence and National Differences in Case Detection

In this cohort of individuals with ARG1‐D at ≤ 18 years of age, we estimate a prevalence of approximately 1:1042080 [95% CI 1:1041580–1:1042581] in Germany, Austria, and Switzerland—consistent with international cohorts [6, 19]. Notably, the prevalence in Austria (1:315666 [1:315174–1:316159]) was nearly fourfold higher compared to the complete DACH region. In contrast, Germany and Switzerland—where ARG1‐D is not part of official national NBS—show lower ascertainment, suggesting that individuals with ARG1‐D may remain un‐ or misdiagnosed [4].

These findings underscore how NBS policies shape epidemiological visibility. Austria's experience provides real‐world evidence that routine screening reduces diagnostic delay, identifies asymptomatic individuals, and may prevent irreversible neurological damage. Despite this potential benefit, ARG1‐D has scarcely been included in NBS programs worldwide as arginine‐based screening accuracy during the first days of life is still debated [6, 20, 21, 22].

### 4.2 Diagnostic Delay and the Impact of Early Detection

Early‐diagnosed patients were identified at a median age of 1 month, while symptomatically diagnosed patients were identified at a median age of 35 months, with a maximum age at diagnosis of 12 years, and a median (range) diagnostic delay of 19 (0–94) months. Symptomatic patients exhibited substantial neurological morbidity, including spastic paraplegia (median onset at 30 months), epilepsy (median onset at 61 months), and dystonia at the time of reporting (median age at reporting: 11.5 years), aligning with established natural history cohorts [1, 12]. In contrast, early‐diagnosed patients displayed minimal/no neurological impairment at the time of study enrollment (median age at reporting: 4 years). Although long‐term outcomes after early diagnosis remain to be evaluated in a larger cohort with age‐matched controls of symptomatically diagnosed participants, this study supports the notion that early detection and treatment improve the disease course. The potential role of emerging treatments such as pegzilarginase—currently approved from the age of 2 years—remains to be established, particularly with regard to long‐term outcomes and use in pre‐symptomatic individuals [9, 10, 13].

### 4.3 ARG1‐D and Its Phenotypical Mimics

ARG1‐D is increasingly recognized as a treatable cause of progressive spastic paraplegia in childhood. Therefore, early diagnosis and differentiation from its clinical mimics, such as childhood‐onset hereditary spastic paraplegias (HSP), hyperornithinemia‐hyperammonemia‐homocitrullinuria (HHH) syndrome, and other urea cycle disorders (UCD) or cerebral palsy (CP), is crucial (Table 2). In contrast to other UCDs, ARG1‐D typically lacks neonatal hyperammonemic crisis and instead manifests with (slowly) progressive lower‐limb spasticity [2, 3, 20, 23, 24]. Therefore, ARG1‐D is frequently misdiagnosed as childhood‐onset HSP or even as CP [4, 5, 27]. Childhood‐onset, complex HSPs—most notably SPG11, SPG15, SPG35, and SPG7—share key neurological features with ARG1‐D, including progressive spastic paraplegia, delayed motor milestones, and gradual functional decline [25, 26]. However, several clinical features strongly favor ARG1‐D over HSPs. First, multisystem involvement is uncommon in HSP but frequent in ARG1‐D, where epilepsy, chronic hepatopathy, and failure to thrive are well‐recognized manifestations. Second, persistent hyperargininemia and episodic hyperammonemia are biochemical hallmarks of ARG1‐D that are absent in HSP [4, 5, 25]. In this cohort, hyperammonemic events occurred in 72% of patients, with comparable peak ammonia concentrations in early‐diagnosed and symptomatically diagnosed individuals, underscoring that metabolic decompensation remains a lifelong risk even after early detection.

**TABLE 2:** Arginase 1 deficiency and its clinical mimics. Key features of arginase 1 deficiency (ARG1‐D), other urea cycle disorders (UCD)–especially hyperornithinemia‐hyperammonemia‐homocitrullinuria (HHH) syndrome, childhood‐onset complex hereditary spastic paraplegias (HSP), and cerebral palsy (CP).

Key feature	Arginase 1 deficiency (ARG1‐D)	Other urea cycle disorders (UCDs)	Hyperornithinemia‐hyperammonemia‐homocitrullinuria (HHH) Syndrome	Childhood‐onset hereditary spastic paraplegias (HSPs)	Cerebral palsy
Age at onset	Typically 2–4 years *This study:* 30 (12–192) months	Variable: neonatal to late‐onset depending on subtype	Childhood to adolescence	Genotype‐dependent (SPG11/15: childhood; SPG35: early childhood; SPG7: adolescence)	Early, < 2 years
Perinatal risk factors for hypoxia	No	No	No	No	Yes
Neurological phenotype					
Spasticity	Yes, progressive, lower limbs; *This study:* 40%	Rare (expect HHH‐syndrome)	Yes, progressive	Yes, progressive	Yes, non‐progressive
Developmental delay	Yes, progressive *This study:* 40%	Yes	Yes, progressive	Variable, mainly complex HSPs	Yes, non‐progressive
Epilepsy	Common	Common	Common	Rare (except SPG11/15)	Variable
Other neurological symptoms	Dystonia, reduced muscle strength, muscular hypotonia	Variable	Ataxia, psychiatric symptoms	Ataxia, dysarthria, extrapyramidal signs, optic atrophy (genotype‐specific)	Variable
Other manifestations					
Hyperammonemia	Yes, usually moderate *This study:* median 170 μmol/L	Yes, recurrent, often severe	Yes, moderate	No	No
Hepatopathy chronic/acute	Possible *This study:* 40%	Possible; acute and chronic	Possible	No	No
Avoidance of high protein food	Common	Common	Common	No	No
Diagnostic features:					
Biochemical hallmarks	Hyperargininemia, episodic hyperammonemia	Recurrent hyperammonemia, amino acids abnormalities	Hyperornithinemia + homocitrullinuria, episodic hyperammonemia	None	None
Screening options	NBS (selected countries), high risk family screening	NBS (selected countries), high risk family screening	NBS (selected countries), high risk family screening	Genetic family screening	None
Confirmatory diagnostic	Plasma amino acids, genetic testing	Plasma amino acids, organic acids in urine, genetic testing	Plasma amino acids, organic acids in urine, genetic testing	Genetic panel testing	Clinical + MRI
MRI pattern	Variable; corticospinal tract changes, mild atrophy	Variable	Variable	Genotype‐specific patterns; e.g., thin corpus callosum, atrophy, white matter changes	Static white matter or basal ganglia injury

*Note:* Based on: [2], [3], [5], [13], [20, 21, 22, 23, 24, 25, 26].

Among urea cycle disorders, HHH syndrome shows the closest phenotypic overlap with ARG1‐D. Both conditions may present with progressive spastic paraplegia, epilepsy, cognitive impairment, and moderate hyperammonemia, often outside the neonatal period [20, 23, 24]. However, HHH syndrome is distinguished biochemically by marked hyperornithinemia and urinary homocitrulline excretion, rather than isolated hyperargininemia. Other urea cycle disorders (e.g., deficiency of ornithine transcarbamylase, argininosuccinate synthetase, or argininosuccinate lyase) may present with chronic neurological impairment but typically show either neonatal onset or recurrent severe hyperammonemic crises, making them less likely mimics of isolated childhood‐onset spastic paraplegia [20, 23, 24].

CP represents another important diagnostic mimic, particularly in young children presenting with early spasticity. However, CP is by definition non‐progressive, with symptoms reflecting static brain injury related to prenatal or perinatal hypoxia, infection, or vascular events [5, 27]. In contrast, ARG1‐D is characterized by progressive neurological deterioration, absence of perinatal risk factors, and the emergence of additional systemic features over time.

Plasma amino acid testing should therefore be performed in all children presenting with unexplained progressive spastic paraplegia, particularly when liver abnormalities, seizures, or developmental concerns coexist.

### 4.4 Limitations

Limitations include small sample size inherent to the rarity of ARG1‐D, cross‐sectional design, and possible reporting bias. The coverage of the “Surveillance Unit of Rare Neurological Disorders in Childhood” is nearly comprehensive in the DACH region, but as participation is not mandatory, underreporting is likely. The higher prevalence observed in Austria might be attributable to newborn screening, suggesting potential underdiagnosis in Germany and Switzerland, but it could also partly reflect overrepresentation of families with more than one affected child, which may disproportionately influence prevalence estimates in extremely rare disorders. Moreover, no patient was eligible for inclusion in arm 2 (unexplained spastic paraplegia). It remains unclear whether this is due to optimal care by neuropediatric centres including amino acid and genetic analysis (exclusion criteria) in all children with unexplained progressive spastic paraplegia or lack of reporting. Moreover, age at reporting differs between early‐ and symptomatically diagnosed participants. Due to pseudonymized data collection and national data protection laws, no genetic data is available for this study. Age‐matched controls and genotype–phenotype correlations are mandatory to discuss whether early diagnosis improves the clinical disease course. This study includes multinational coverage, both early‐ and late‐diagnosed patients, and a detailed phenotyping using a harmonized data set, but longitudinal follow‐up of age‐matched cohorts, including treatment responses to pegzilarginase, will be essential to define modern natural history.

## 5 Conclusions

This study confirms the broad clinical spectrum and low prevalence of ARG1‐D of approximately 1:1000000 newborns in the DACH region. Newborn and high‐risk family screening reduce the age at diagnosis and enable an early start of therapy in asymptomatic individuals. Thus, early screening offers the potential to improve outcome compared to individuals diagnosed after symptom onset.

## Author Contributions

S. Scharre, A. L. Hess, U. Muetze, S. Kölker conceptualized and designed the study; S. Scharre, A. L. Hess coordinated patient data acquisition; S. Scharre, A. L. Hess, D. Karall, A. Baghdasaryan, M. Huemer, J. B. Hennermann, A. Hahn, M. Lindner, G. Gramer, T. Luecke, N. Kleinelanghorst, J. Haeberle, A. S. Lotz‐Havla carried out data collection; J. G. Okun, P. Feyh managed laboratory analysis; F. Gleich, S. Scharre, A. L. Hess carried out data management; S. F. Garbade and S. Scharre carried out statistical analysis; S. Scharre, S. F. Garbade, F. Gleich, U. Muetze, S. Kölker evaluated and interpreted data; S. Scharre, U. Muetze, S. Kölker prepared the manuscript; All authors revised and reviewed the manuscript for important intellectual content. All authors approved the final manuscript as submitted.

## Funding

The study was generously supported by Immedica Pharma AB, Solnavägen 3H, SE‐113 63 Stockholm (to S. Kölker). The content of the article has not been influenced in any way by the sponsor.

## Ethics Statement

The study was approved by the local ethics committee in Heidelberg (S‐675/2023, PI: S. Kölker). The study was conducted in accordance with the ethical standards of the responsible committee on human experimentation (institutional and national) and with the Helsinki Declaration of 1975, as revised in 2013.

## Conflicts of Interest

S. Scharre received travel funds from Immedica Pharma A. B., U. Muetze received speakers honoraria from Synlab. M. Huemer has received unrestricted research grants from Nutricia Metabolics, Sanofi, Travere and consultancy honoraria or travel support from Chiesi, Nutricia Metabolics, Sanofi, Recordati, and Immedica Pharma in the past 36 months. A. S. Lotz‐Havla received honoraria for educational events (Vitaflo), meeting and travel support (Nutricia, PTC Therapeutics), and honoraria for advisory boards (Immedica, PTC Therapeutics). G. Gramer received meeting and travel support (Nutricia, Biomarin Pharmaceutical Inc.), honoraria for advisory boards (iECURE, Moderna), and research grants (Biomarin Pharmaceutical Inc.). Anna Baghdasaryan received honoraria for participation in advisory boards and travel support form Immedica Pharma A. B., A. Hahn received speakers honoraria from Immedica Pharma A. B., J. B. Hennermann received honoraria and/or travel expenses from Amicus, Chiesi, Immedica, Takeda, and Sanofi. All other authors declare no conflicts of interest.

## Supporting information


**File S1:** Case report form. The study‐specific case report form contained information on medical history, clinical symptoms, diagnosis and treatment and was provided in German with English translation.

## Data Availability

Research data are not shared.
