# Supplementary material for: Prevalence, Disease Onset and Clinical Outcome in Arginase 1 Deficiency: Cross‐Border Surveillance in Germany, Austria, and Switzerland
Source: J Inherit Metab Dis. 2026 Jun 7;49(4):e70210. doi: 10.1002/jimd.70210 (PMC13243979; doi:10.1002/jimd.70210)
Supplement: Supplementary file 1 — File S1: Case report form. The study‐specific case report form contained information on medical history, clinical symptoms, diagnosis and treatment and was provided in German with English translation. [file JIMD-49-0-s001.pdf]

Epidemiologie und präzise Phänotypisierung des Arginasemangels durch gezielte Diagnostik symptomatischer Patientinnen und Patienten

1) Stammdaten / *demographic data*

Geburtsjahr / *year of birth* ________________ ☐ Unbekannt/ *unknown*

Geschlecht / *sex* ☐ w / *f* ☐ m ☐ d ☐ Unbekannt

Konsanguinität der Eltern / *parental consanguinity* ☐ Nein / *no* ☐ Ja / *yes* ☐ Unbekannt

Familienmitglieder mit gleicher Krankheit / *family members with same disease:*
☐ Nein / *no* ☐ Ja / *yes* ☐ Unbekannt/ *unknown*

Hauptdiagnose(n) (gerne ICD-10) / *main diagnosis(es) (ICD-10):* ______________________________

____

Perinatalperiode / *perinatal period*:

Gestationsalter / *gestational age*: ____________________ ☐ Unbekannt/ *unknown*

Geburtsgewicht / *birth weight*: ____________________ g

(V.a.) Asphyxie/Hypoxie / *suspected asphyxia/hypoxia:* ☐ Nein / *no* ☐ Ja / *yes,* ☐ Unbekannt/ *unknown*
APGAR: ____/____/____, pH ___

Neonatale Infektion/Sepsis / *neonatal infection/sepsis:* ☐ Nein / *no* ☐ Ja / *yes* ☐ Unbekannt/ *unknown*

Intra-/Periventrikuläre Hämorrhagie / *intra-/periventricular hamorrhage*:
☐ Nein / *no* ☐ Ja / *yes*, Grad /*grade* I☐ II☐ III☐ IV☐ ☐ Unbekannt/ *unknown*

2) Diagnose / *diagnosis*

Bestätigte Diagnose eines Arginasemangels: ☐ Ja, weiter bei **a)** ☐ Nein, weiter bei **b)** ☐ Unbekannt / *unknown*
Confirmed diagnosis of ARG1-D: *Yes, continue with **a)*** *No, continue with **b)***

a) Konfirmation durch / *confirmation by*: ☐ Unbekannt / *unknown*
☐ Biochemische Analyse / *biochemical*
☐ Enzymatische Analyse / *enzymatic*
☐ Molekulargenetische Analyse / *genetic*

Diagnosemodus / *mode of diagnosis:*
Selektive Diagnostik nach Auftreten von Symptomen / *selective diagnostic*
Diagnostik als Geschwister eines bekannten Indexpatienten / *high-risk family screening*
Sonstiges / *other*: ______________________________ ☐ Unbekannt / *unknown*

Alter bei Diagnose / *age at diagnosis:* ________________________ Monat/e / *month(s)* ☐ Unbekannt / *unknown*

➔ **weiter bei Abschnitt „Behandlung" / *continue with section „treatment"***

b) Erfolgt eine Behandlung mit oder Substitution von L-Arginin: ☐ Nein ☐ Ja ☐ Unbekannt / *unknown*
Is treatment with or substitution of L-arginine being performed: *no* *yes*

Wurde für diese/n Patient/in bereits eine biochemische (Bestimmung der Aminosäuren im Plasma oder Trockenblut) oder molekulargenetische Diagnostik (Sanger-Sequenzierung des *ARG1*-Gens bzw. Exom-/Genomsequenzierung) durchgeführt? *Has biochemical diagnostics (amino acids in plasma or dried blood spots) or molecular genetic diagnostics (Sanger sequencing of the ARG1 gene or exome/genome sequencing) already been performed for this patient?*
☐ Nein / *no* ☐ Ja / *yes* ☐ Unbekannt / *unknown*

→ Wurde in Abschnitt b) zweimal mit Nein geantwortet, können wir kostenfrei eine Trockenblutkarte des/r Patienten/in analysieren / *If "No" was answered twice in section b), we can analyze a dried blood spot card from the patient free of charge*

→ **Weiter bei Abschnitt „Neurologische Symptome" / continue with section "neurological symptoms"**

3) Behandlung bei bestätigtem Arginasemangel / *treatment in confirmed ARG1-D patients*

Protein-reduzierte Diät / *protein-restricted diet*: ☐ Nein/*no* ☐ Ja/*yes* ☐ Unbekannt / *unknown*

Stickstofffänger (Natriumbenzoat, Natrium-/Glycerolphenylbutyrat) / *nitrogen scavengers (sodium benzoate, sodium/glycerol phenylbutyrate)*: ☐ Nein/*no* ☐ Ja/*yes* ☐ Unbekannt

Enzymersatztherapie (Pegzilarginase) / *enzyme therapy*: ☐ Nein/*no* ☐ Ja/*yes* ☐ Unbekannt

Lebertransplantation / *liver transplantation*: ☐ Nein/*no* ☐ Ja, im Alter von / *yes, at the age of* ___________ Monaten / *months* ☐ Unbekannt

4) Neurologische Symptome / *neurological symptoms*

Progressive, spastische Para-/Tetraplegie / ***progressive**, spastic paraplegia*: ☐ Nein/*no* ☐ Ja,/*yes,* ☐ Unbekannt / *unknown*

☐ untere Extremität /*lower extremity* ☐ obere Extremität / *upper extremity,* GMFCS level: _______

Alter bei Beginn der spastischen Para-/Tetraplegie / *age at onset*: ___________ Monat/e / *month/s* ☐ Unbekannt

Orthopädische Hilfsmittel / *orthopedic aids*: ☐ Nein/*no* ☐ Ja/*yes* ☐ Unbekannt

Ataxie / ataxia: ☐ Nein/*no* ☐ Ja/*yes* Tremor: ☐ Nein/*no* ☐ Ja/*yes* Chorea: ☐ Nein/*no* ☐ Ja/*yes* ☐ Unbekannt / *unknown*

musk. Hypotonie / *musk. hypotonia*: ☐ Nein/*no* ☐ Ja/*yes* Dystonie / *dystonia:* ☐ Nein/*no* ☐ Ja/*yes*

red. Kraftgrad / *red. muscle strength:* ☐ Nein/*no* ☐ Ja/*yes*

Epilepsie / *epilepsy*: ☐ Nein / *no* ☐ Ja / *yes* ☐ Unbekannt / *unknown*

Alter bei Diagnose Epilepsie / *age at diagnosis*: _________________ Monat/e / *month/s*

Verlust von Fähigkeiten / *loss of skills*: ☐ Nein/*no* ☐ Ja/*yes* ☐ Unbekannt / *unknown*

(Globale) Entwicklungsverzögerung / *(global) developmental delay*: ☐ Nein/*no* ☐ Ja/*yes* ☐ Unbekannt / *unknown*

Entwicklungs-/Kognitionstestung erfolgt / *cognitive testing performed*: ☐ Nein/*no* ☐ Ja/*yes*

Alter bei letzter Testung / *age at last assessment*: ___________________ Monate / *months*

Test: ☐ BSID ☐ Denver ☐ WPPSI ☐ WAIS ☐ WISC ☐ Sonstiges /*other*:

____ ☐ Unbekannt / *unknown*

Global IQ/ Mental Development Index (MDI): _________________________________

5) Metabolische Entgleisung/en / *metabolic decompensation/s*

Bestätigte Hyperammonämie / *confirmed hyperammonemia*: ☐ Nein/*no* ☐ Ja/*yes,* max.: __________ (☐ µmol/l / ☐ µg/dl) ☐ Unbekannt / *unknown*

Gab es jemals umschriebene Episoden mit / *have there ever been episodes of*:

Vigilanzminderung / *reduced vigilance*: ☐ Nein/*no* ☐ Ja/*yes*

Unklarem Erbrechen / *unexplained vomiting*: ☐ Nein/*no* ☐ Ja/*yes* ☐ Unbekannt / *unknown*

Nahrungsverweigerung / *food refusal:* ☐ Nein/*no* ☐ Ja/*yes*

Akuter Leberfunktionsstörung / *acute liver dysfunction:* ☐ Nein/*no* ☐ Ja/*yes* (AST, ALT, INR ↑)

Gang-/Gleichgewichtsstörung / *gait/balance disorder*: ☐ Nein/*no* ☐ Ja/*yes*

Kopfschmerzen / *headaches*: ☐ Nein/*no* ☐ Ja/*yes*

Freier Kommentar / *free comment*: ____

Falls ja, vorangegangener möglicher Auslöser/*if yes, possible preceding trigger*: ☐ Nein/*no* ☐ Ja,/*yes,* ☐ Unbekannt / *unknown*

____

6) Weitere Symptome / *additional symptoms*

Gedeihstörung / *failure to thrive*: ☐ Nein/*no* ☐ Ja/*yes* ☐ Unbekannt / *unknown*

Größe /*height* (Perzentile): _______________

Gewicht / *weight* (Perzentile): _______________

Mikrozephalie / *microcephaly*: ☐ Nein/*no* ☐ Ja, aktuelle Perzentile KU/*yes, current head circumference percentile:* _________________ ☐ Unbekannt / *unknown*

Psychiatrische Symptome / *psychiatric symptoms*: ☐ Nein/*no* ☐ Ja/*yes,* ☐ Unbekannt / *unknown*

____

Chron. Leberfunktionsstörung / *chronic liver dysfunction*: ☐ Nein/*no* ☐ Ja (Hepatomegalie/GOT, GPT oder INR ↑ >3 Monate)/ *yes (hepatomegaly/AST, ALT or INR ↑ for longer than 3 months)* ☐ Unbekannt / *unknown*

Andere Symptome (+Alter bei Beginn (Monat/e)) / *Other symptoms (+ age at onset (month/s)):* ☐ Unbekannt / *unknown*

____
